# Women’s satisfaction in early versus delayed postcaesarean feeding: A one-blind randomized controlled trial study

**Published:** 2015

**Authors:** Shahnaz Barat, Sedigheh Esmaeilzadeh, Masoumeh Golsorkhtabaramiri, Soraya Khafri, Maryam Moradi Recabdarkolaee

**Affiliations:** 1 Obstetrics and Gynecology Department, Babol University of Medical Sciences, Babol, Iran.; 2 Fatemezahra Infertility and Reproductive Health Research Center, Babol University of Medical Sciences, Babol, Iran.; 3 Social Medicine Department, Babol University of Medical Sciences, Babol, Iran.; 4 Ayatollah Rouhani Hospital, Babol University of Medical Sciences, Babol, Iran.

**Keywords:** Caesarean section, Satisfaction, Gastrointestinal, Feeding

## Abstract

**Background:**

The early postoperative feeding after caesarean section (C- section) has remained controversial. This study was designed to evaluate the safety and efficacy of early versus delayed postcaesarean section oral feeding regarding gastrointestinal complications and patients postoperative satisfaction after C- section.

**Methods:**

This clinical trial study was conducted on 200 pregnant women undergoing planned C-section under spinal anesthesia (Registration Number: IRCT: 138712211760N1). Women were randomly divided in two groups; group A (early feeding group) comprised of 101 patients who were encouraged to take oral ﬂuid. If they tolerated, they continue semi-solid and solid foods starting 2 h after caesarean section. Group B (delayed feeding group) comprised of 99 patients who were given oral ﬂuid 8 h after surgery. After beginning of feeding the patients’ tolerance, first flatus, first defecation, beginning of regular diet, the length of hospital stay and also patient satisfaction level were evaluated in each group by visual scale analog (VAS).

**Results:**

The mean time of the ﬁrst passage of ﬂatus was 10.2±1.7 hours for the early oral feeding group versus 10.7±1.6 hours for the delayed feeding group and the difference was significant (P=0.03). Duration to first defecation and length of hospital stay as well as patient satisfaction level did not differ significantly between the two groups.

**Conclusion:**

The results of this study suggest early postcaesarean feeding. It is well tolerated and helps return normal feeding habits.

Caesarean section (C-section) is the most common operation for women and although it is a short-term surgery with less manipulation, however, it needs precaution care. The characteristics features of postoperative ileus include abdominal distension and lack of passage of flatus or stool. These signs worsen at post-partum uterine contractions. Prolonged hospitalization, increases the risk of hospital infections, decreases patient satisfaction and increases health care costs and other complications (1, 2). Recently, the necessity of the post C-section delayed feeding has been under question (3, 4). Unfortunately, in many hospitals, the patients are routinely prohibited of oral feeding at least 8 h after C-section or until the return of bowel sounds and the passing of flatus. Some earlier studies showed internal feeding needs hemodynamic stability.and is associated with complications (5). They reported starting nutrition within 24 h after surgery is ideal, but conventional manner is within 48 h (6).

Recently, some studies have shown that early postoperative oral feeding after C-section does not enhance gastrointestinal complications. (7-9). Reduction in the duration of hospitalization in order to diminish the costs of therapy and improving the rotation of hospital beds associated with minimum global health threatening is the goal of contemporary surgeries (1, 10-12). One of the main concerns of any woman surgeon is the earlier return of the patients to normal feeding habits after caesarian section. This study was designed to evaluate the effect of early versus delayed postcaesarean feeding on gastrointestinal function and patient postoperative satisfaction after discharge. 

## Methods

289 healthy pregnant women undergoing elective C- section under spinal anesthesia were enrolled in this randomized, single blind trial study. 223 women were recruited and randomized to the treatment according to our criteria. All operations were carried out at Ayatollah Rouhani Hospital of Babol University of Medical Sciences from May 2010 to April 2011. The Ethics Committee of Babol University of Medical Sciences approved the study. 

Inclusion criteria included: singleton pregnant women undergoing planned C- section under spinal anesthesia. Exclusion criteria included women with a history of bowel surgery, maternal disease and intraoperative or immediate postoperative major complications, fetal anomaly, gastrointestinal diseases, intestinal surgery, intestinal adhesion, obstetrics complications (such as fatty liver in pregnancy, preeclampsia, placental abruption, chorioamnitic membrane, the presence of severe adhesions, major blood loss , and the occurrence of surgical complications, diabetes, user of the tocolytic drugs and also who had emergency C- section under general anesthesia. was excluded to create homogenous study population to obtain more reliable data. Following-up of the study and explaining potential complications to all enrolled women, individually contributed in the study. All enrolled women were the same regional analgesic regimen for C- section with 2 ml lidocaine 5% and 25 mcg/kg fentanile as needed for the relief of pain. It is notable that all women had fasting 6 h prior to the elective operation (13). In addition, all women underwent a transverse incision (pfannenstiel) in the site of C-section. In addition, a carbohydrate drink 2hr prior operation has important limitation. Each enrolled woman undergoing C- section was randomized by using a computer-generated sequence concealed from the obstetrician and the statistical consultant. The surgeons (investigators) were blinded to the study group. Group A (delayed feeding group) comprised of patients who were encouraged to oral ﬂuid (semi solid and solid) starting 2 h after C- section was performed. Group B (early fed group) comprised of patients who received oral ﬂuid at 8 h following surgery. After C-section, the patient’s demographic information such as age, body mass index (BMI), duration of surgery, time to bowel movement, first flatus, first defecation, beginning of a regular regimen, the length of hospital stay, patient tolerance of oral feeding and regular diet, vomiting in patients and the satisfaction level was recorded by a nurse.

Satisfaction rates of our patients were evaluated with visual analogue scale (VAS) (7, 14, 15). It has also been accepted as a tool to measure quality of life (15). A VAS ranging from zero to 10 was selected to represent the postoperative satisfaction level. The zero on the ruler was aligned with the lack of satisfaction and ten represented full satisfaction. Accordingly, the observed categories were collapsed into a 1-10 rating scale. Factors which often contribute to postoperative hospital discharge after planned C- section under spinal anesthesia are considered as; absence of fever for at least 24 hours, onset alienating of the fluids and intravenous medications and patient's tolerance of regular diet, passage of flatus, lack of pain or tolerance of pain with oral analgesics (16). The sample size was estimated by G power 3. Statistical analysis of data was performed using the two-tailed hypothesis with significance set at a p-value of ≤0.05. T- tests and chi-square tests were used to determine the relationship between postoperative gastrointestinal complications and early feeding. 

## Results

**Figure 1:**
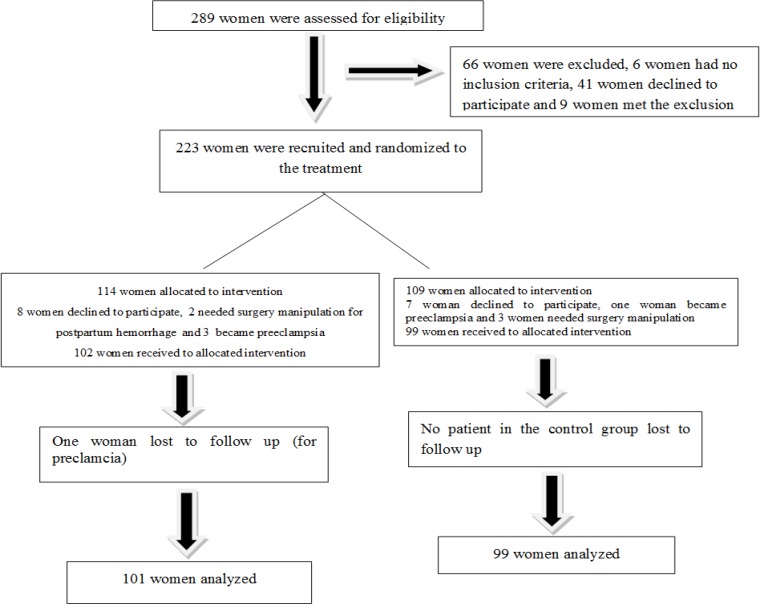
Patients from recruitment to completion of early versus delayed postcaesarean feeding

289 women underwent elective C- section. 66 women were excluded. 223 women were recruited and randomized to treatment. 114 women were allocated to the early intervention group and 102 women. 109 women were allocated to delayed-fed intervention group and 99 women completed the study. One of these women lost to follow-up (for preeclampsia) in the early-fed group. No patients in the control group lost to follow up. 101 women in early-fed group and 99 women in delayed-fed group were analyzed (figure 1).

The mean age and body mass index of participants in the early fed group was 27.85±5.18 years and 30.74±3.57 kg/m^2^, respectively. The mean duration of surgery in women was 38.15±4.85 minutes. There were no differences between the two groups in demographic and clinical characteristics of the patients (table 1). 

**Table1:** Demographic criteria of the two groups under study

**Criteria**	**Early feeding** **(Mean±SD)**	**Delayed feeding** **(Mean±SD)**	**Pvalue**
Age(yr)	27.85±5.18	26.96±5.22	0.42
BMI(Kg/m^2^)	30.74±3.57	30.88±3.53	0.77
Surgery duration(min)	38.15±4.85	36.65±5.55	0.75

* Sig: P<0.05

Fluids were allowed in the two study groups after passing of ﬂatus. From the first hour postoperative oral intake the early oral feeding group after C- section did not appear to have any gastrointestinal complications. 

The mean time to the ﬁrst passage of ﬂatus was 10.2±1.7 hours for the early oral feeding group and 10.7±1.6 hours for the delayed feeding group with a significant difference (P=0.03). 

The patient satisfaction level (by VAS) as well as time to the first bowel movement, ﬁrst defecation, and length of hospital stay demonstrated improvement although no different significance was shown between two groups. One patient in the early-fed group had postoperative vomiting and was treated with a single dose of intravenous antiemetic medicine (table 2). 

**Table 2:** Postcaesarean gastrointestinal complications in the early and delayed feeding groups

**Criteria**	**Early feeding** **(n=100)**	**Delayed feeding** **(n=100)**	**P-value**
vomiting up to 1 hr after feeding (times)	1	0	1
vomiting up to 2 hr after feeding (times)	0	0	1
Time to bowel sound(hr) (mean ±SD)	100	100	1
Time to passage of ﬂatus(hr) (mean ±SD)	10.19±1.71	10.70±1.58	0.03*
Time to defecation (mean ±SD)(hr)	24.73±1.27	24.79±1.26	0.74
Time to start regular diet(hr)	21.7±4.08	22.3±2.2	0.16
Hospital stay (day) (mean ±SD)	45.6±15.81	46.56±13.56	0.65
Satisfaction level (VAS)(mean ±SD)	83.15±4.58	82.15±4.88	0.14

* Sig: P<0.05

## Discussion

Our findings showed that the early postoperative oral feeding is well tolerated as well as delayed feeding. In addition, in this group the time to the first passage of ﬂatus was lower compared with the delayed postoperative oral feeding. The findings are consistence with Gocmen et al.’s study (17). It is noteworthy that bowel movements intensify postdelivery pain (1) and early passage of flatus improves it fast. The evidence of safety and usefulness of the early postoperative oral feeding after major operation such as C- section, intestinal resection or anastomosis, and even intestinal perforation and peritonitis are reachable (4, 18, 19).

In our query as well as some studies, the gastrointestinal complications in early-fed group were compared with the delay-fed group but the incidence of complications in their study was more in our study in each group (7, 20). Most probable reasons were as follows: first, we included only planned C- section and excluded the patients with unsuccessful induction of labor or received sedation). Second, applying 6 hours fasting before the operation for the patients with elective C-section to prepare their digestive system. Third, is due to our method of anesthesia that was spinal and may cause illeus itself. Moreover, all of our patients received our standard routine antiemetic prophylaxis for C-section which sluggish bowel motility.

All the above conditions caused some limitations in selecting adequate samples. In Teoh et al study which is similar to our study, the population study in the planned C-section was performed by spinal anesthesia represented the lower incidence of gastrointestinal complications which was 3% while in another prospective study proportion of complication was 26 to 31% (7). The main difference of our study with other studies is; was the time duration considered as the onset of delayed feeding (8 h after the operation) while this duration was considered as early feeding in other studies (11, 20-23). Satisfaction is a complex physiological and multidimensional response (24). Satisfaction rates of our samples obtained following early feeding after C-section were compared to those obtained following delayed feeding. Some research reported a greater degree of satisfaction in the early-fed group (22).

We also investigated the duration of stay in the hospital. Our result showed that the length of hospital stay was shorter in the early-fed group but not significantly. Perhaps that is because of the inadequate sample size in our study. In many other studies, the early feeding has been associated with either the decreased length of stay in hospital (16, 21, 12) or a greater tendency to hospital discharge (18, 7). Teoh believed it improves the economic policies of his country (7). 

The earlier discharge from hospital has a positive effect on the recovery process as well as cost-effectiveness (9). Considering the advantages and disadvantages of treatment, researchers should rely on clinical outcomes as well as patients' preferences to propose further practical solutions. We suggest a well-designed clinical trial study that evaluates the safety and benefits of early feeding in emergency C- section.

In conclusion** e**arly feeding after (planned) uncomplicated C-section is safe and well-tolerated as well as in delayed feeding. It quickly leads to normal feeding habits. Hence, facilitating discharge to the women after caesarian section.

## Notes

**Funding: **This research was financially supported by the Vice-Chancellery for Research of Babol University of Medical Sciences.
